# Exploring Tactile Temporal Features for Object Pose Estimation during Robotic Manipulation

**DOI:** 10.3390/s23094535

**Published:** 2023-05-06

**Authors:** Viral Rasik Galaiya, Mohammed Asfour, Thiago Eustaquio Alves de Oliveira, Xianta Jiang, Vinicius Prado da Fonseca

**Affiliations:** 1 Robotics and AI Lab, Department of Computer Science, Memorial University of Newfoundland and Labrador, St. John’s, NL A1C 5S7, Canada; 2 Ubiquitous Computing and Machine Learning Lab, Department of Computer Science, Memorial University of Newfoundland and Labrador, St. John’s, NL A1C 5S7, Canada; 3 Haptics and Robots Research Group, Department of Computer Science, Lakehead University, Thunder Bay, ON P7B 5E1, Canada

**Keywords:** tactile sensing, object manipulation, LSTM, sliding window, pose estimation

## Abstract

Dexterous robotic manipulation tasks depend on estimating the state of in-hand objects, particularly their orientation. Although cameras have been traditionally used to estimate the object’s pose, tactile sensors have recently been studied due to their robustness against occlusions. This paper explores tactile data’s temporal information for estimating the orientation of grasped objects. The data from a compliant tactile sensor were collected using different time-window sample sizes and evaluated using neural networks with long short-term memory (LSTM) layers. Our results suggest that using a window of sensor readings improved angle estimation compared to previous works. The best window size of 40 samples achieved an average of 0.0375 for the mean absolute error (MAE) in radians, 0.0030 for the mean squared error (MSE), 0.9074 for the coefficient of determination (R${}^{2}$), and 0.9094 for the explained variance score (EXP), with no enhancement for larger window sizes. This work illustrates the benefits of temporal information for pose estimation and analyzes the performance behavior with varying window sizes, which can be a basis for future robotic tactile research. Moreover, it can complement underactuated designs and visual pose estimation methods.

## 1. Introduction

Many areas of human activity, such as mass-production factories, low-invasive surgeries, and prostheses, have adopted robotic manipulation systems. Robotic manipulation is exceptionally reliable when the system has complete information regarding the environment. These systems usually must follow a set of trajectories, interact with objects of known features, and perform repetitive tasks with minimal environmental adaptation, which limits the use of manipulation systems to performing activities in unstructured settings. Recent advancements in data-driven methods, innovative gripper design, and sensor implementation have reduced the limitations of robotic manipulators in such environments. Nevertheless, there are hurdles to the applications of robotic arms in unstructured environments or dexterous tasks such as the complex manipulation of daily objects [1].

One main challenge is estimating the object’s orientation during the aftergrasp phase. The object’s orientation can change from an initial visual estimation due to calculation errors, external forces, finger occlusion, and clutter. After a successful grasp, one approach is to use tactile sensors to extract object information, improving the object’s pose estimation.

Robotic hands have immense flexibility despite their use in specific domains, such as prostheses, with limitations regarding the human hand’s size, weight, and shape. By sacrificing the initial stability and uncertainty in the grasp pose estimation, an underactuated approach substantially reduces the planning time and gripper design complexity [2]. However, it is fundamental for robotic arms to estimate the handled object’s pose so that they operate optimally in object manipulation applications. For instance, the grasp used by a gripper of a robotic arm or a prosthesis to hold a mug might change if its handle is at a different angle.

Object orientation estimation depends on several aspects, such as the gripper’s configuration, the sensors used, and how the data are analyzed. Different sensors are used in robotic manipulation to categorize the properties of an object, such as its orientation. Pregrasp poses are commonly obtained using computer vision [3]. However, visual data alone can be insufficient as the gripper approaches the object and the range of occlusion increases. This limitation is particularly pronounced when the camera’s location is fixed or under unpredictable circumstances, i.e., in unstructured environments. For instance, using a top-view camera to estimate the object pose is not feasible for an arm prosthesis, whereas prosthesis-mounted cameras are susceptible to occlusion. Moreover, once the gripper grasps the object, it will cover at least part of it, making it difficult to estimate its orientation. Furthermore, merely using vision cannot reduce the forces and related environmental stimuli, leading to potential errors in the estimation of the orientation due to a miscalculated geometry, friction, forces, camera occlusion, and clutter [1,4].

Due to the limitations of visual methods, several applications use tactile sensing [5,6,7,8,9,10] while grasping the object, providing more relevant information that is not interrupted [11]. Tactile sensing has shown promise in specific use cases, such as in minimally invasive surgery [12] or cable manipulation [13], and is also being shown to be a good supplement to control system optimization [14,15]. Sensors such as pressure sensors [10], force sensors [16], and inertial sensors [17] are gradually becoming more prevalent for object pose estimation and object recognition. In addition, tactile sensors provide aftergrasp contact information about the object that can be used for control [13] or in-hand manipulation. Nevertheless, there have also been developments of vision-based tactile sensors ranging from using internal reflection [18,19] to observing the deformation of the surface [20].

Tactile sensing can be a vital link for overcoming computer vision limitations and can result in a better performance of robotic manipulation. Previous works used machine learning models and visual frames of reference to train models that learned the aftergrasp object angle, which was later used to estimate the object’s pose [21]. However, previously seen data can affect the current estimation of the object’s state. Thus, estimating the current object pose can be improved by considering temporal data, such as in sliding-window sampling. For this reason, in the present work, we study the effect of temporal data based on sliding-window sampling to train a deep learning model for object angle estimation.

## 2. Literature Review

Orientation estimation has been a part of pose estimation in robotics research for a long time. Recent studies have made leaps regarding orientation estimation with sufficiently low error due to advancements in sensor technology, most importantly tactile sensors [22,23].

For instance, Ji et al. [24] proposed a novel model-based scheme using a visual–tactile sensor (VTS) [25]. In their study, the sensor compromised a deformable layer that interacted with objects with a depth camera behind the said layer to generate a depth map of the deformation caused by the object. They reported orientation errors for three objects of under $3^{\circ }$. However, detecting their objects’ rotations could have been visually easier compared to more uniform smooth shapes such as cylinders or ellipsoids.

Additionally, Suresh et al. [26] formulated the tactile sensing problem as a simultaneous localization and mapping (SLAM) problem, in which the robot end effector made multiple contacts with the object to determine its pose. They reported a rotational root-mean-square error (RMSE) of 0.09 radians. However, their method assumed the initial pose and scale of the object roughly and it neglected factors outside the controlled setup that might change the object’s orientation.

Other studies utilized information about the robot arm alongside tactile data to estimate the orientation of objects. Alvarez et al. [27] used the kinematic information and a particle filter for the pose estimation via tactile contact points, force measurements, and the angle information of the gripper’s joints. Their algorithm initiated a pose estimation using visual data, which was refined by a particle filter based on the optical data. After experiments with three objects of different sizes, they reported an error of $0.812^{\circ }$ in their best experiment, which rose to $3.508^{\circ }$ in their worst case. Results aside, the method required a known kinematic model of the robotic arm and a top-view camera for inference, which is infeasible in some applications, such as daily activities using prostheses.

To relax the requirement of a detailed kinematic model, recent research has explored underactuated grippers while relying on machine learning methods to build a model of the object pose. For instance, Azulay et al. [28] conducted a wide-scope study to investigate objects’ pose estimation and control them with underactuated grippers. They incorporated haptic sensors, joint angles, actuator torques, and a glance at the pose at the start of the gripper’s movement. Using the robotic arm’s kinematic model, they concluded that some combinations of the tested features were better suited for object manipulation than others. They reported a root-mean-square error (RMSE) of $3.0\pm 0.6^{\circ }$ for the orientation using a neural network with LSTM layers, their best model. Using multiple features alongside the kinematic model can require a more significant computational ability than processors on end devices alone, such as prostheses, during daily activities.

However, investigating orientation estimation in itself outside practical uses can limit the reported results in some situations. For instance, robotic grippers that handle objects can occlude the object partially or fully, affecting visual-based approaches. Furthermore, objects can rotate during handling due to many factors, such as slipping or external forces, thus requiring methods to estimate the objects’ orientation during the grasp phase.

High-density tactile sensors, akin to the human hand, are another direction that can provide much information. Funaabashi et al. [29] used graph convolution neural networks (GCNs) to extract geodesic features from three-axis tactile sensors across 16 degrees of freedom of a robotic hand providing 1168 measurements at 100 Hz. They used eight objects with two different hardness, slipperiness, and heaviness factors. They compared various GCN configurations and a multilayer perceptron, and the GCN model with the most convolution layers was the best performer. The limitation of this method was due to the need for a large number of computational resources and sensors, and the ambiguity of intermediate states, although accounting for different properties, such as hardness and slipperiness, improved the possibilities of generalization.

To develop a solution that required minimal finger path planning, a relaxed kinematic model requirement, and a less-needed processing of images, Da Fonseca et al. [21] developed an underactuated gripper with four compliant sensing modules on flexible fingers, and investigated the collected sensor data while grasping objects of three distinct sizes. The experiments included a top-view camera to obtain a visual frame of reference for the ground-truth orientation. The method used the tactile sensors’ information to represent the object angle, whereas the ground-truth angle was obtained from the camera frame. Finally, the authors compared five regression models trained using tactile data to estimate the object’s angle. The best models reported by the authors were the ridge regression model and linear regression, obtaining a $1.82^{\circ }$ average mean square error. The authors used random data sampling for model training in the paper and left possible relationships among the time-series samples as a future research point. Still, given that the tasks were dynamic, they expected that the near samples in the time-series sensor data would be correlated with the angle.

Some studies also investigated the fusion of tactile and visual data for orientation estimation during object handling.

Alvarez et al. [30] proposed a fusion method of the visual data and tactile data to estimate the object’s pose during grasp. A camera tracked the object during grasp, whereas a particle filter was utilized with the tactile data to reduce the uncertainty of the object’s pose. They reported that their method obtained an orientation error varying from $1^{\circ }$ to $9.65^{\circ }$. Their method yielded a high variance of the estimation error, in addition to requiring a 3D model of the handled object for the method to be used.

Dikhale et al. [31] proposed the sensor fusion of visual and tactile data as well. Their method used neural networks to process the tactile and visual data separately before fusing them to give a final prediction of the object’s pose. They reported an angular error as small as $3^{\circ }$; however, it reached a high of $24^{\circ }$, showing a high variance in the estimation depending on the object.

From the previous studies, we find that different factors affect the orientation estimation performance and eligibility. For instance, computationally demanding methods, such as the ones relying on inverse kinematics or particle filters, are inappropriate for small devices, such as prostheses limited to an onboard processor, whereas relying on visual data, solely or with sensor fusion, is prone to occlusion during the grasp phase as top-view cameras are not feasible in many applications. Hence, a model must only estimate the object’s angle using tactile data during grasp, without kinematics, to reduce computation while providing an acceptable angle error.

In this paper, we evaluate the use of sliding-window sampled tactile data to estimate the yaw angle under the stable grasp of an object while relaxing the kinematic model requirement by using an underactuated gripper and a compliant bioinspired sensing module that includes magnetic, angular rate, gravity, and pressure sensing components. We analyze the temporal nature of tactile signals by using a neural network that contains long short-term memory (LSTM) layers to estimate the orientation with the highest precision for objects. The models trained in the present work were based on Da Fonseca et al. [21], taking in a window of readings from the sensors mounted on the gripper and then outputting the estimated object’s orientation at the end of this sampling window. As the paper’s main topic is the in-hand orientation estimation, our method uses only the initial grasp orientation as a reference and does not require information from the gripper joints, its kinematics model, a multitude of sensors, or during-grasp visual data.

Our method can be utilized in a multitude of applications from everyday use to factory settings due to its dependency on only a small number of tactile sensors without the need for additional types of sensors. Furthermore, our method does not need computationally capable machines as it utilizes only a neural network that can run on a computational device as small as a flash drive, such as Google Coral, due to advancements in computational technology. In addition, the proposed method’s performance is not prone to uniform shapes whose orientation change is hard to determine visually, such as rotating cylinders.

## 3. Materials and Methods

Here, we describe the data collection and preprocessing methods used for the sliding-window sampling tactile data, the models trained for the experiments, and how we organized the sampling strategy for pose estimation.

### 3.1. Data Collection

We used tactile data collected in a previous study [21] from an underactuated gripper with two independently controlled fingers during object-grasping tasks to evaluate the sliding-window sampling strategy for pose estimation.

**Figure 1:**
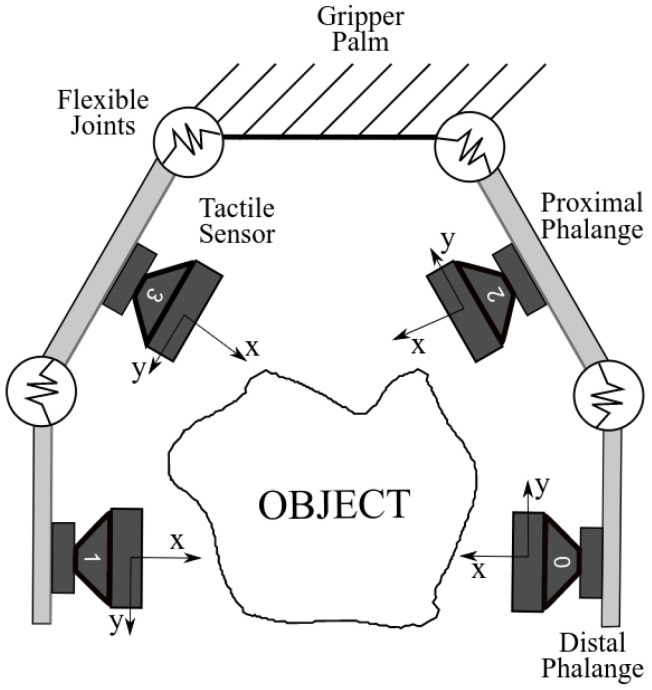
The underactuated gripper [21] diagram with two fingers, each with two phalanges and their respective sensors.

In the gripper developed by Prado da Fonseca et al. [21], each phalanx has a fixed tactile sensor developed by Alves de Oliveira et al. [17], as shown in Figure 1.

**Figure 2:**
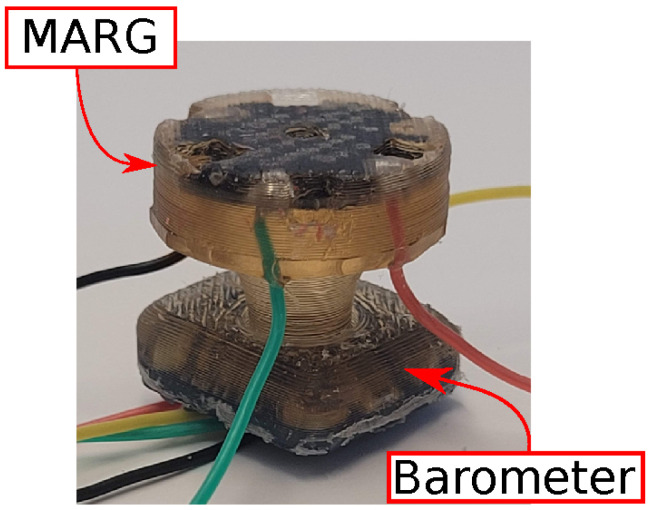
The sensor with its base attached to the manipulator and close to where the barometer is, and its surface over the MARG sensor is in contact with the object.

Each sensor provides deep pressure information from a barometer in addition to angular velocity, linear acceleration, and magnetic field in all three axes using the nine-degree-of-freedom magnetic, angular rate, and gravity (MARG) system. The barometer, as shown in Figure 2, is encased in a polyurethane structure close to the base, and the MARG sensor is placed closer to the point of contact so it can detect microvibrations. The fabrication structure of the sensor enables the pressure to be transferred from the contact point to the barometer effectively. The compliant sensor structure allows the contact displacement to be measured by the inertial unit while the deep pressure sensor measures the contact forces. The data are collected using an onboard microcontroller interfacing via I2C with a computer running the Robot Operating System (ROS) framework [32].

**Figure 3:**
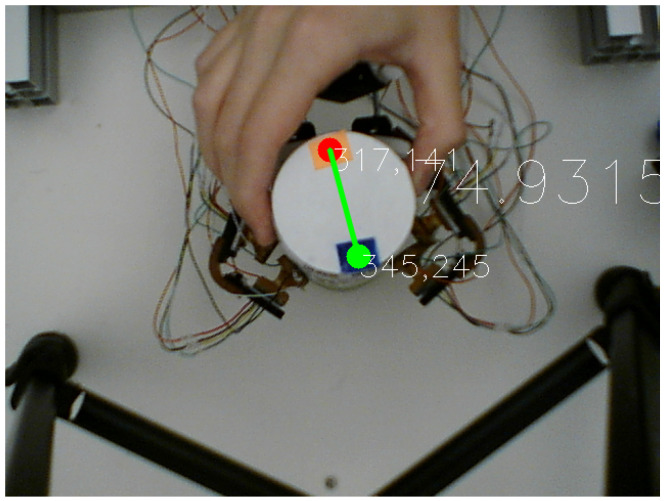
The object’s two markers to obtain the ground-truth angle using computer vision [21].

Prado da Fonseca et al. [21] used the allocentric reference frame from the camera pointed down to calculate the object’s angle. The top-view angle of the object was extracted using two colored markers attached to it to identify key points using the OpenCV library as shown in Figure 3.

The angle between the two markers line and the fixed camera frame horizontally in the clockwise direction was established to be the object’s angle, and the object was considered at $90^{\circ }$ on the *x*-axis. These points were later compared to a fixed frame of reference at the camera’s center to determine the object position change relative to the specified frame of the gripper. The stable grasp was obtained using a dual fuzzy controller that obtained microvibrations and pressure feedback from the tactile sensor [33]. This procedure was performed with three cylindrical objects with 57 mm, 65 mm, and 80 mm diameters. The objects were rotated manually in the CW and CCW directions, simulating external forces causing the object to change its orientation during grasp. Although this motion was at a low speed, the human element of this motion provided inconsistent forces, which the model was able to take into account to provide an accurate prediction. Such movements also simulated the act of parasitic motions, which are undesired motion components leading to a lower manipulation accuracy/quality [34], despite being in a stable grasp. Moreover, the three different objects were used to determine the ability of the model to generalize among similar objects. The ground truth angle after rotation was obtained relative to the form of reference from the top-view camera as seen in Figure 3.

### 3.2. Data Characteristics

The preprocessing methods used in this work depended highly on the time-series details of the data available from Prado da Fonseca et al. [21]. For instance, the number of instances in each window sample could be affected by the different frequencies of each sensor. Table 1 shows the average sampling frequency of each sensor, where the slowest sensor is the camera, ranging from 9 to 29.95 Hz. The fastest sensor is the MARG sensor, ranging from 911.33 to 973.50 Hz.

**Figure 4:**
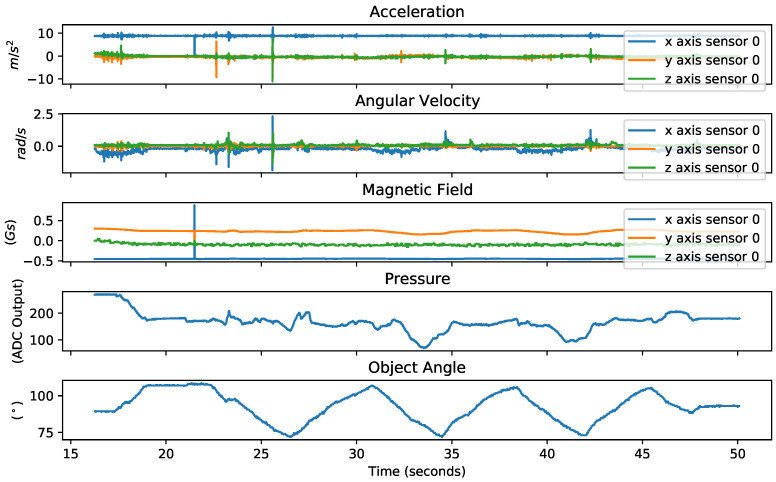
The angle of rotation, the corresponding pressure, and one of the four MARG outputs in one of the trial data collection trials.

As mentioned in Section 3.1, the data collection consisted of a CW and CCW rotation procedure performed by an external operator on three cylindrical objects with 57 mm, 65 mm, and 80 mm diameters. The dataset for each object contained sensor readings from 5 different operations of external rotation. Figure 4 shows the disturbances of rotation on the pressure, linear acceleration, angular velocity, and magnetic field for one sensor in relation to the angle during external rotations.

The data characteristics described here are sufficient for our investigation. Further details about the data collection protocol and attributes can be found in the original data collection study [21].

### 3.3. Preprocessing

**Figure 5:**
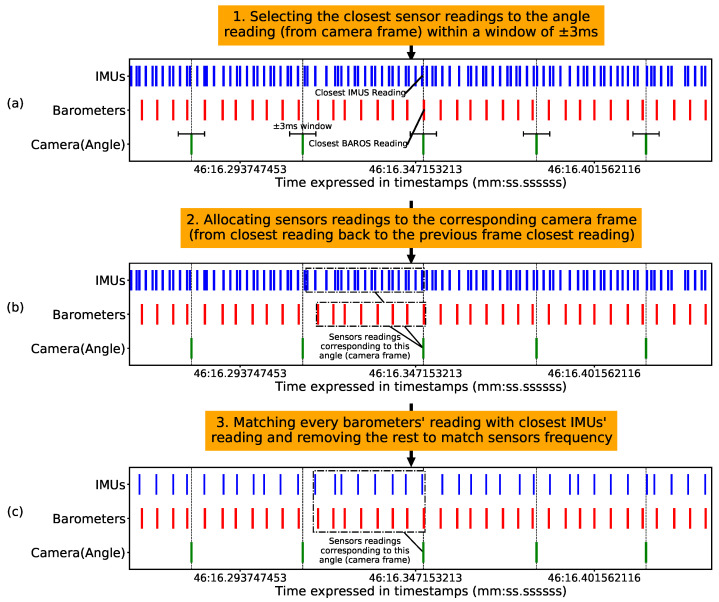
The procedure used for sensors alignment. (**a**) For every angle determined by the camera, the closest corresponding pressure and MARG values were selected. (**b**) Sensor values were grouped with the corresponding camera frame. (**c**) MARG values were downsampled to match the frequency of the pressure sensor.

The listener ROS node collected data at different time instances since the camera, MARG sensor, and pressure communicated asynchronously. Therefore, the signals needed to be aligned for our strategy of window sampling. First, we scaled the data to utilize deep learning methods. Subsequently, to add LSTM layers, we had to reconcile the sampling frequency differences for the various sensors by synchronizing and downsampling their data. Since the lowest frequency was the camera frames, their timestamps acted as a reference for our procedure of sensor alignment presented in Figure 5. Afterward, we reshaped the data to incorporate the previous states for each instance of the ground truth angle.

Figure 5a shows that the obtained pressure and MARG signals were within three milliseconds from the angle from the camera frame, on average. Figure 5b shows the MARG and pressure values collected between two camera frames to correspond to a single frame. Finally, since the pressure was sampled at a lower frequency than the MARG sensor, Figure 5c shows the MARG sensor reading closest to the corresponding pressure reading was kept, and the remaining samples in between the selected ones were discarded. The whole process is summarized in the Algorithm 1.
**Algorithm 1** Preprocessing and experimentation pseudocode1: **for** each barometer reading **do**:2:      keep closest MARG reading3:  Discard the rest of the MARG reading4: **for** For each angle value **do**:5:      take sensor readings of corresponding timestamp6:      take (WindowSize−1) previous sensor readings7:  Separate training and test data8:  Normalize training and test sensor values using the mean and standard deviation from training data9:  Train model using training data10:  Obtain performance results using test data


In this approach, small window sizes would only utilize signals corresponding to the selected camera frames. In contrast, overlapping with signals corresponding to previous frames was used to obtain more data for large window sizes.

After alignment, the final dataset contained five runs for each of the three object sizes. Each run consisted of 900 camera frames, which had an average of 8 corresponding samples from the sensors per frame. We used the data for all object sizes to ensure the dataset size was sufficient for model training. Since all the sensors had different magnitudes and distributions, all the data apart from the object angle were scaled. Finally, we standardized the rest of the dataset. We use the following equation to normalize each sensor’s data.

(1) $$N^{\left(i\right)}=\frac{X^{\left(i\right)}-\mu^{\left(i\right)}}{\sigma^{\left(i\right)}}$$

where $N^{\left(i\right)}$ is the standardized signals of the *i*th sensor. $X^{\left(i\right)}$ are the raw signals of the *i*th sensor, $\mu^{\left(i\right)}$ is the mean signal value of the *i*th sensor, and $\sigma^{\left(i\right)}$ is the signal’s standard deviation.

### 3.4. The Angle Estimation Model

Since tactile sensing measurements from objects under grasp manipulation are continuous and sequential, we used time-series-based neural networks, specifically long short-term memory (LSTM)-based networks, to analyze the window sampling.

#### 3.4.1. Model Architecture

Using a small baseline model initially, we arrived at the final model after adding layers that provided the best marginal improvement in performance for its size without overfitting, as increasing the model’s size overfitted the training data.

**Figure 6:**
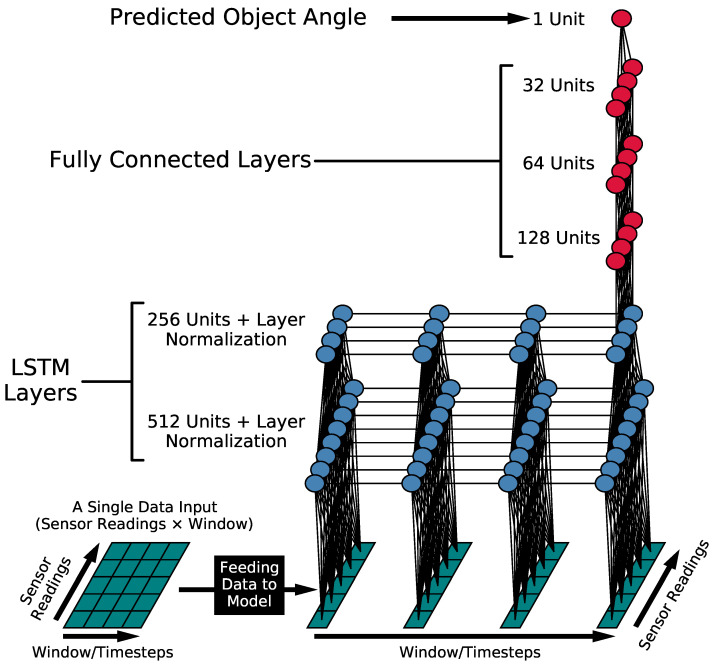
The model architecture and the feed-forward of a single input sample through the model. The architecture consists of 2 LSTM layers with normalization layers with 512 Units and 256 units, respectively, and 3 fully connected layers with 128, 64, and 32 neurons.

Figure 6 shows the final model architecture we established consisting of two LSTM layers with normalization layers with 512 units and 256 units, respectively, and three dense fully connected layers with 128, 64, and 32 neurons, respectively. All of the experiments were conducted on Compute Canada, an advanced research computing platform, using the Python programming language and Tensorflow [35] library to preprocess the data and train the model.

We used the mean absolute error (MAE) between the angle’s estimated and actual values as the training loss function. Moreover, we chose MAE as it diminishes in value much slower than a mean square error (MSE) as the model’s estimation gets closer to the actual angle and has a value of less than one.

#### 3.4.2. Hyperparameters and Window Size Optimization

Various experiments were performed to provide an understanding of the data and identify the effects of hyperparameters and the performance corresponding to their variations. In particular, we manipulated batch sizes and windows and explored regularization methods. We explored the trade-off between window size and performance based on the best results to find the best gain in accuracy for a small model size. This trade-off is fundamental in mobile robotics, with less memory and computational time leeway. We performed a grid search to determine the hyperparameters over the number of epochs, learning rate, and batch size. We chose the best configuration of hyperparameters to conduct the study and investigate the window sampling technique. We used a cross-validation with four folds, with six iterations for the model per fold, to ensure the consistency of the model’s performance and report any variance in the metrics scores. Table 2 shows the neural network hyperparameters.

## 4. Results

Here, we present the results of our experiment to estimate in-hand objects’ orientation using a sliding-window sampling strategy and evaluate it with LSTM models. The evaluation metrics used were the mean squared error (MSE), mean absolute error (MAE), coefficient of determination (R${}^{2}$), and explained variance score (EXP).

### 4.1. Model Training

**Figure 7:**
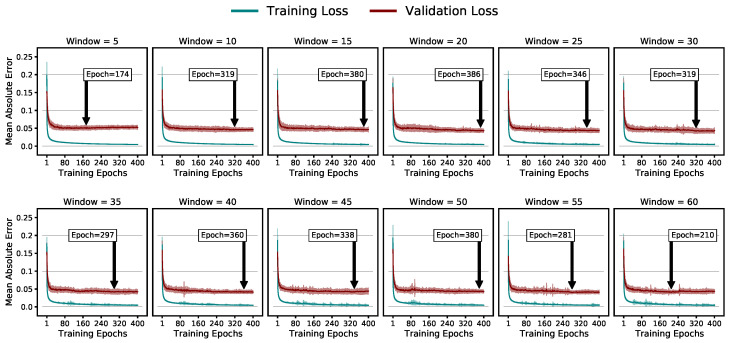
Average training and validation losses and their standard variation for the different window sizes, highlighting the average epoch of the lowest validation error.

Figure 7 depicts the training and validation losses during the training phase while highlighting the average epoch of the lowest validation error averaged over the folds and model iterations.

We prevented overfitting by training the models for 400 epochs and selecting the model weights at the epoch of the lowest validation loss.

### 4.2. Window Size

**Figure 8:**
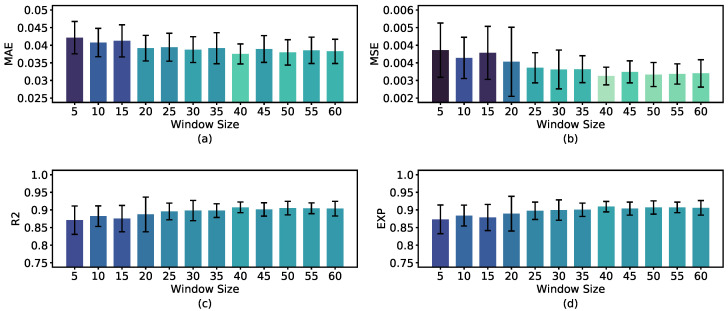
The performance results from varying the window size. (**a**) Mean Absolute Error (MAE). (**b**) Mean Squared Error (MSE). (**c**) Coefficient of determination (R${}^{2}$ Score). (**d**) Explained Variance (EXP).

The primary factor of temporal data explored by this paper was the window size. Figure 8 shows that a window size of 40 achieved the lowest error. It revealed a performance improvement as the window size expanded; however, the improvement magnitude decreased asymptotically.

The above result indicated that a window of 40 samples effectively captured the necessary amount of tactile information for estimating the object’s orientation, regardless of the metric used. Larger window sizes, beyond 40 samples, did not result in any further improvement in model performance. This finding is further supported by Table 3.

The model achieved the best MAE of 0.0375 radians with a window of 40 and an average error of 0.0408 with a window size as small as 10 samples. The model also obtained high R${}^{2}$ and EXP scores of 0.9074 and 0.9094, respectively, for the best window size.

**Figure 9:**
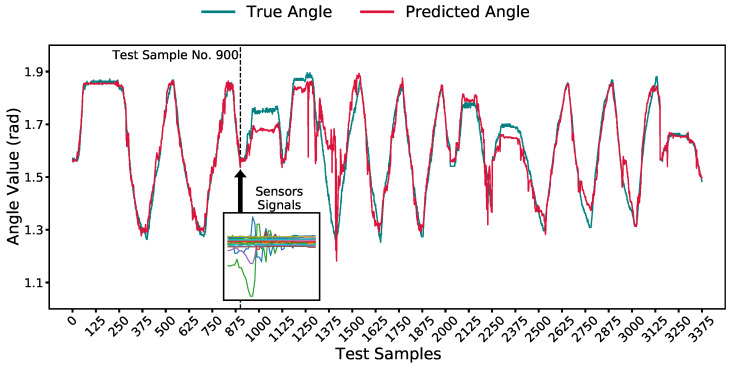
A comparison of the predicted and ground-truth angle for one of the iterations of the model with the best window size of 40 samples. A 40-sample window corresponding to the prediction of the angle for sample 900 is highlighted.

We used one of the iterations of the best model to illustrate its angle prediction compared to the ground truth in Figure 9. The figure also shows a window of 40 samples of sensors’ readings that correspond to a single angle prediction, test point no. 900.

### 4.3. Comparing this Temporal Deep Learning Method to Ridge Regression

Nevertheless, we trained linear and ridge regression models with the same data protocol we applied for the neural networks for comparison. Notably, these two classifiers presented the best results in an approach that did not use the time-series relation in the date [21], in which the models were trained per object size and not using all the sizes at once. Although we cannot conclude the advantage of temporal data from the MAE and MSE values due to different normalization and scaling procedures’ ranges, the R${}^{2}$ and EXP scores highlighted that point in the previous study [21]. The results of these two models are reported in Table 4 using our preprocessing procedure for comparison.

## 5. Discussion

This study aimed to determine if pose estimations related to the time-series tactile data captured by the sliding-window sampling strategy adopted. We analyzed the performance of using a neural network with LSTM layers in estimating the angle of the handled object by a tactile-sensing robotic hand, considering different sliding-window sizes of input samples. The deep learning model was compared to standard regression models to showcase the improvement due to their temporal tactile data incorporation.

We presented a data processing procedure to align the collected data from multiple asynchronous sensors and approximate their readings’ timestamps to yield multisensor temporal data in Figure 5. The data were then used to train and evaluate a deep learning model, whose architecture we optimized, as shown in Figure 6, and whose optimal training hyperparameters were found using a grid search.

By testing a range of window sizes between 5 and 60 to investigate the degree of impact of the temporal relation between tactile data, we demonstrated the importance of such relations between sensor readings for estimating the angle of an object under grasp. We found that incorporating a small window size of five inputs gave an acceptable performance of 0.0422 radians, equivalent to 2.417 degrees, and scores above 0.87 for both the R${}^{2}$ and EXP metrics. Compared to the standard classifiers tested in this study, we found that the smallest window could improve the R${}^{2}$ and EXP scores by about 26% and 24%, respectively, and could give a reduction of 0.0256 and 0.0047 for the MAE and MSE, respectively. Thus, it showed that the temporal relationships of the sensor readings could improve the estimation of the objects’ angle, as evident in Table 3 and Table 4.

Furthermore, these results gradually improved by integrating more sensor information from larger window sizes of up to 40 samples per window, after which the performance saturated. Including more past readings beyond 40 samples did not add valuable information to the instantaneous angle value prediction as seen in Figure 8. This result showed that despite the importance of temporal relationships in tactile data for estimating the object’s angle during manipulation, these relationships diminished asymptotically after a threshold.

For the best window size of 40 samples, we found that it achieved an acceptable error for many applications with an average of 0.0375 for the MAE in radians, and it could explain most of the variance in the distribution, shown in the 0.9074 R${}^{2}$ score and 0.9094 EXP score. This performance is sufficient in multiple applications without a camera reference during the grasp phase, thus supporting the use of temporal tactile data for orientation estimation of in-hand objects in unstructured environments.

Notably, the model could achieve such results after training on data from objects with differing sizes, thus incorporating more variation in the data, making the temporal relation harder to capture, therefore improving on our previous results [21], where only a per object angle estimation was performed. Additionally, using different object sizes also generalized the model performance. This generalization also extended to being applied in an underactuated system which experienced larger effects of parasitic motions (compared to fully actuated systems). However, this was a limited application that did not account for the other dimensions, and, as a result, future work can include all other axes, provide a complete object pose description, and improve the robustness. In addition, we could not directly compare the metrics because of different normalization methods, as they used a normalized degree unit, whereas we used radians.

Future research can use our results as a reference and investigate a tactile dataset with objects of different shapes as well as remaining degrees of freedom to determine the complete change in the object’s pose, not only its yaw orientation. Moreover, feature engineering can be an additional step alongside the temporal tactile data to enhance the model further. Future studies can benefit from the proposed alignment of asynchronous sensors that we illustrated in Figure 5.

Finally, collecting a dataset of both arm-mounted and gripper-mounted tactile data for object orientation estimation can further illustrate the benefits of temporal tactile sensing compared to other techniques.

## 6. Conclusions

This paper illustrated the importance of temporal tactile data in estimating the orientation of in-hand objects by proposing a model architecture with LSTM layers that used signals from tactile sensors on the fingers. We evaluated these experiments’ performance using the MAE, MSE, R${}^{2}$, and EXP metrics. The results showed that including temporal data benefited the orientation estimation of the objects up to an asymptotic threshold, as investigating a range of window sizes concluded that the smallest window studied boosted the performers compared to standard regression models, such as linear and ridge regression. The best window size in the investigated range was 40 input samples, which could predict the object angle with an average MAE of 0.0375 radians. Our model also had an R${}^{2}$ value of 0.9074 and an EXP value of 0.9074, respectively. By comparison, the ridge regressor yielded an average MAE of 0.0677 radians, an R${}^{2}$ score of 0.6875, and an EXP value of 0.7033. Therefore, the relationship between the tactile signals and the object’s angle was better explained with time-series models that utilized the temporal relationships of the sensors’ readings. These results highlight the benefits of using previous state information, particularly because manipulation tends to be sequential. At the same time, it presents a simple architecture that uses less processing and computational power compared to setups with high-density tactile sensors. Moreover, our tactile data model can work with objects such as symmetric cylinders that may look fixed from the visual sensors’ perspective. Finally, it also presents the viability of pose estimation without needing 3D models. Our proposed model can be included in future research investigating the pose estimation problem using tactile data and the importance of their temporal relations with different modes of pose change. Future studies can also benefit from our proposed preprocessing procedure to match the timestamps of readings obtained from asynchronous sensors.

## Figures and Tables

**Table 1:** The average frequency of the data obtained from its respective sensors.

Camera	Pressure	MARG Sensor
29.95 Hz	402.19 Hz	973.50 Hz

**Table 2:** The hyperparameters’ values of the neural network.

Hyperparameter	Value
Learning rate	0.00025
Batch size	128
Epochs	400
K-folds	4
Iterations	6

**Table 3:** The detailed results of the inspected window size range using MAE and MSE errors in radians, R${}^{2}$ score, and EXP.

Window	MAE	MSE	R${}^{2}$	EXP
5	0.0422 ± 0.0046	0.0042 ± 0.0012	0.8710 ± 0.0404	0.8732 ± 0.0408
10	0.0408 ± 0.0040	0.0038 ± 0.0009	0.8823 ± 0.0292	0.8840 ± 0.0297
15	0.0412 ± 0.0046	0.0041 ± 0.0012	0.8754 ± 0.0374	0.8785 ± 0.0370
20	0.0392 ± 0.0036	0.0036 ± 0.0016	0.8873 ± 0.0492	0.8894 ± 0.0493
25	0.0394 ± 0.0040	0.0034 ± 0.0007	0.8956 ± 0.0237	0.8975 ± 0.0246
30	0.0388 ± 0.0037	0.0033 ± 0.0009	0.8981 ± 0.0287	0.8997 ± 0.0289
35	0.0392 ± 0.0044	0.0033 ± 0.0006	0.8981 ± 0.0193	0.9005 ± 0.0188
40	0.0375 ± 0.0028	0.0030 ± 0.0004	0.9074 ± 0.0153	0.9094 ± 0.0148
45	0.0389 ± 0.0038	0.0032 ± 0.0005	0.9013 ± 0.0190	0.9038 ± 0.0185
50	0.0380 ± 0.0036	0.0031 ± 0.0005	0.9053 ± 0.0192	0.9069 ± 0.0188
55	0.0385 ± 0.0037	0.0031 ± 0.0005	0.9048 ± 0.0153	0.9073 ± 0.0149
60	0.0383 ± 0.0034	0.0031 ± 0.0006	0.9037 ± 0.0208	0.9060 ± 0.0207

**Table 4:** The results of standard regression models using MAE and MSE errors in radians, R${}^{2}$ score, and EXP.

Model	MAE	MSE	R${}^{2}$	EXP
Ridge regressor	0.0677	0.0088	0.6875	0.7033
Linear regressor	0.0678	0.0089	0.6862	0.7021

## Data Availability

Data sharing not applicable.
